# Developmental and evolutionary origins of the pharyngeal apparatus

**DOI:** 10.1186/2041-9139-3-24

**Published:** 2012-10-01

**Authors:** Anthony Graham, Jo Richardson

**Affiliations:** 1 MRC Centre for Developmental Neurobiology, King’s College London, London SE1 1UL, UK

**Keywords:** Deuterostomes, Endoderm, Operculum, Parathyroid, Pharyngeal arches, Pharyngeal pouches, Vertebrate evolution

## Abstract

The vertebrate pharyngeal apparatus, serving the dual functions of feeding and respiration, has its embryonic origin in a series of bulges found on the lateral surface of the head, the pharyngeal arches. Developmental studies have been able to discern how these structures are constructed and this has opened the way for an analysis of how the pharyngeal apparatus was assembled and modified during evolution. For many years, the role of the neural crest in organizing pharyngeal development was emphasized and, as this was believed to be a uniquely vertebrate cell type, it was suggested that the development of the pharyngeal apparatus of vertebrates was distinct from that of other chordates. However, it has now been established that a key event in vertebrate pharyngeal development is the outpocketing of the endoderm to form the pharyngeal pouches. Significantly, outpocketing of the pharyngeal endoderm is a basal deuterostome character and the regulatory network that mediates this process is conserved. Thus, the framework around which the vertebrate pharyngeal apparatus is built is ancient. The pharyngeal arches of vertebrates are, however, more complex and this can be ascribed to these structures being populated by neural crest cells, which form the skeletal support of the pharynx, and mesoderm, which will give rise to the musculature and the arch arteries. Within the vertebrates, as development progresses beyond the phylotypic stage, the pharyngeal apparatus has also been extensively remodelled and this has seemingly involved radical alterations to the developmental programme. Recent studies, however, have shown that these alterations were not as dramatic as previously believed. Thus, while the evolution of amniotes was believed to have involved the loss of gills and their covering, the operculum, it is now apparent that neither of these structures was completely lost. Rather, the gills were transformed into the parathyroid glands and the operculum still exists as an embryonic entity and is still required for the internalization of the posterior pharyngeal arches. Thus, the key steps in our phylogenetic history are laid out during the development of our pharyngeal apparatus.

## Review

While ontogeny does not simply recapitulate phylogeny, it is undoubtedly true that ontogeny is shaped by phylogeny. Developmental processes have evolutionary histories and these can be uncovered through experimental analysis and comparative studies across a range of species. It is through these approaches that insights into how developmental processes have been assembled over evolution can be garnered. In this article, we wish to discuss the development of the pharynx and to make the case that this process has been profoundly shaped by its evolutionary history. Importantly, we are now at a point where the developmental and evolutionary studies can be brought together and we can identify steps that have emerged successively during evolution. We can uncover deeply conserved features of pharyngeal development that preceded the emergence of the vertebrates and indeed can now be seen to have evolved as early as the deuterostomes. We can also relate the remodelling of the pharyngeal region that occurs during development to evolutionary modifications that occurred within the vertebrates. We would argue that in the pharynx developmental events collectively betray our phylogenetic history.

### The pharyngeal arches

**Figure 1:**
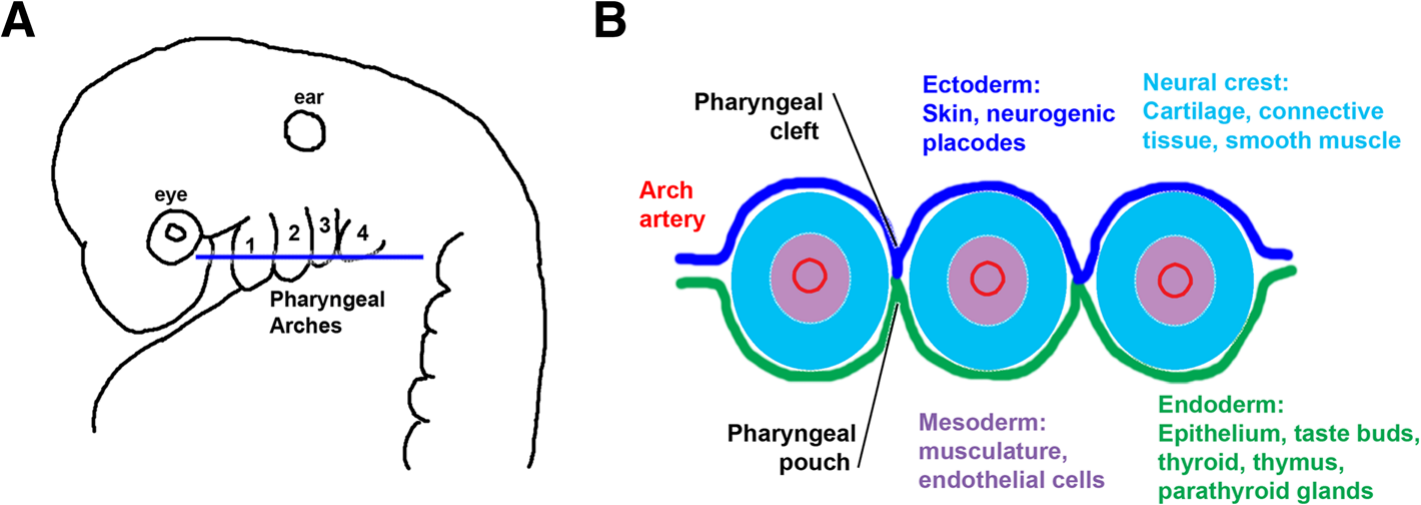
**The vertebrate pharyngeal arches and their derivatives. **(**A**) Lateral view of an amniote embryo, showing the characteristic bulges of the pharyngeal arches number 1 to 4 from anterior. The pouches intercalate between the arches. The position of the eye and ear are shown. (**B**) Schematic of a transverse section through the arch region, showing the constituent tissues: ectoderm, dark blue; endoderm, green; neural crest, pale blue ; mesoderm, purple.

Although it is not readily apparent when considering adult anatomy, our pharyngeal apparatus has a metameric origin, arising from a series of bulges found on the lateral surface of the head of the embryo, the pharyngeal arches. These structures are first evident at about three to four weeks of human development, and it is within these that the nerves, muscles, skeletal tissues and epithelial specializations of the pharynx are subsequently laid down and fashioned. The development of these structures is, however, complex and involves interplay between a number of disparate embryonic populations (Figure 1) [1]. The ectoderm, which lies externally, will give rise to the epidermis and form localized thickenings, termed neurogenic placodes, the sensory neurons that will innervate the pharynx. Internally, the endoderm forms the lining of the pharynx, as well as a number of specialized organs: the thyroid, parathyroids and thymus. Lying between these two layers are the cells that fill the arches, the mesoderm and the neural crest. The mesoderm, which lies centrally within the arches, forms the endothelial cells of the arch arteries and the musculature, while the neural crest cells that surround the mesoderm will form the skeletal and connective tissues. Between the arches, the ectoderm and the endoderm contact each other and thus demarcate the anterior and posterior boundaries of each arch. This is evident externally as the ectodermal clefts, and internally as the endodermal pouches.

**Figure 2:**
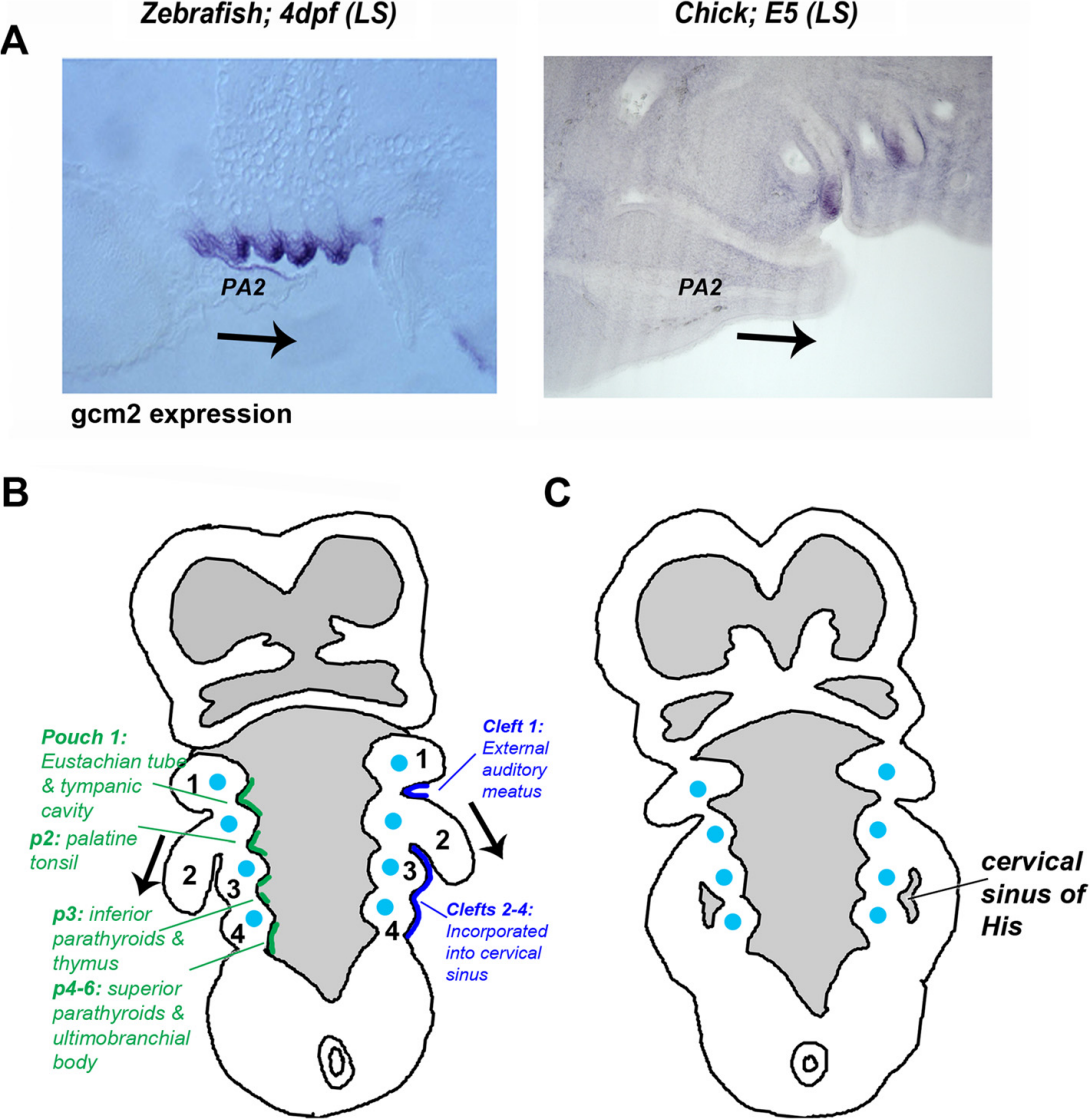
**Internalization of the posterior pharyngeal arches in amniotes. **(**A**) Expression of *gcm2 *in zebrafish and chicks at comparable stages. In fish, this transcription factor is expressed in the pharyngeal pouches and their derivatives, the gill buds, and is required for their development. In chicks, *gcm2 *is also expressed in the pharyngeal pouches, which subsequently give rise to the parathyroids (modified from [3]). (**B**) Schematic of a transverse section through a human embryo, showing the second arch growing caudally over the posterior pouches (arrows). Internally, the pouches form several structures (indicated in green) derived from the endoderm. Ectodermal derivatives are indicated in dark blue. The posterior end of the pharynx is closed. (**C**) Later in development, the expanding second arch fuses with the body wall, enclosing the posterior arches and pouches. A sinus is formed, which is later obliterated. (**B** and **C**) adapted from Larsen [2].

As development progresses, this relatively simple metameric organization becomes obscured. The first arch forms the jaw but the more posterior arches become involved in a complex rearrangement that results in their obliteration. This process is initiated by the caudal expansion of the second arch, which grows to cover the more posterior arches (Figure 2B). The caudal edge of the second arch subsequently fuses with the underlying epithelium at the level of the cardiac eminence (heart protrusion), which results in the posterior pharyngeal arches becoming enclosed in a cavity, named the cervical sinus of His, which eventually becomes obliterated by the apposition and fusion of its walls yielding the smooth contour to the external surface of the neck [2].

### The development of the pharyngeal arches - a key role for endoderm

Experimental studies in the twentieth century in a number of vertebrate model systems highlighted the importance of the neural crest in directing arch development [4-6]. It is from the neural crest cells that the skeletal elements of the arches derive; heterotopic transplantation of neural crest cells was shown to result in skeletal transformations. However, it was subsequently shown that neural crest cells play a less pervasive role than previously believed and that the endoderm is a major player in organizing pharyngeal development. The first indication of pharyngeal arch formation is not the migration of neural crest cells from the brain but rather the outpocketing of the endoderm to form the pharyngeal pouches [7,8]. Ablation studies in chicks also demonstrated that the pharyngeal pouches will form and contact the ectoderm in the absence of neural crest cells, and that these crestless pharyngeal segments are regionalized and have a sense of identity [7]. For example, in the absence of neural crest cells the second pharyngeal arch is still marked by a high level of *Shh* expression at its posterior margin [7]. Furthermore, in the zebrafish *vgo* mutant, crest migration is normal but the posterior pharyngeal endoderm fails to segment and form the pouches, and consequently there is a failure in the normal development of the posterior pharyngeal arches [9]. The importance of endodermal outpocketing in defining arch number is also apparent during normal development. In all vertebrates, there is a single post-otic stream of neural crest cells that fills a variable number of posterior arches - seven in lampreys, five in teleosts, three in amniotes - that emerge and are defined after the formation of the pharyngeal pouches [10-12].

The emphasis on the central role played by the neural crest in vertebrate pharyngeal development also distracted attention from key conserved features of this developmental programme that predate the emergence of the vertebrates. Neural crest cells had previously been viewed as being a defining vertebrate feature [13] and thus the key role of neural crest cells in organizing the development of the pharyngeal arches in vertebrates seemed to underline the distinctiveness of the vertebrate pharynx from that of other chordates. However, the fact that it is the outpocketing of the endoderm that underpins pharyngeal arch formation opened up broader avenues for comparison in non-vertebrate chordates.

### Deuterostome origins of endodermal outpocketing

In cephalochordates, such as amphioxus, there are no neural crest cells to fill the pharyngeal region and the pharyngeal slits are relatively simple; perforations form at the points of contact between the ectoderm and the endoderm and these pharyngeal segments are supported by an endodermally secreted acellular cartilage [14]. The pharyngeal slits assist in filtering food particles from the water; these are extensive, covering some 30% to 50% of the length of the animal.

Homology between the formation of these gill slits and pharyngeal pouch formation in vertebrates can be assessed via an analysis of the expression of amphioxus orthologues of key players in the development of the vertebrate pharyngeal pouches. Prominent amongst these are a *Pax-Six-Eya* regulatory network, and mutational analyses in mice support a model in which *Eya1* acts upstream of *Six* genes in the pharyngeal endoderm [15,16]. *Eya1* and *Six* proteins are also known to interact and it is suggested that these factors positively regulate the expression of *Pax1* within the pouches [15,16]. Significantly, as is seen in vertebrates, the amphioxus *Pax1/9*, *Eya*, *Six1/2* and *Six4/5* genes are all coexpressed in the pharyngeal endoderm [17,18]. In vertebrates, *Tbx1* is another gene that plays a key role in driving the outpocketing of the pharyngeal endoderm. This gene is expressed in the pharyngeal pouches and mesoderm of the arches and mutations in *Tbx1* result in a failure to generate the posterior pharyngeal pouches, and in amphioxus *Tbx1/10* gene is similarly expressed in the pharyngeal segments [19]. Thus, the expression domains of these orthologues of key pharyngeal genes provide strong evidence for homology between pharyngeal development in vertebrates and amphioxus.

Given that the presence of a series of pharyngeal slits is a defining chordate feature, homology between vertebrate pharyngeal pouches and amphioxus pharyngeal perforations is perhaps to be anticipated. However, it has also become clear that pharyngeal development built around endodermal outpocketing is more ancient and that it is probably a deuterostome characteristic. It was shown a number of years ago that hemichordate gill slits also express the *Pax1/9* gene [20] and more recently a comprehensive analysis of pharyngeal slit formation in *Saccoglossus kowalevskii* provided further strong evidence for homology between hemichordate gill slits and vertebrate pharyngeal pouches [21]. In this species, it was similarly observed that *Pax1/9*, *Eya* and *Six* expression is associated with the formation of the gill pores by the endoderm. *Tbx1* expression, however, was not found to be associated with the pharyngeal endodermal. Of course, it should be noted that the other major deuterostome clade, the echinoderms, lack gill slits. This, however, is a result of a secondary loss and paleontological evidence has shown that the earliest echinoderms were bilateral and did possess gill slits [22].

### Construction of the vertebrate pharyngeal arches - the influence of endoderm on the neural crest

**Figure 3:**
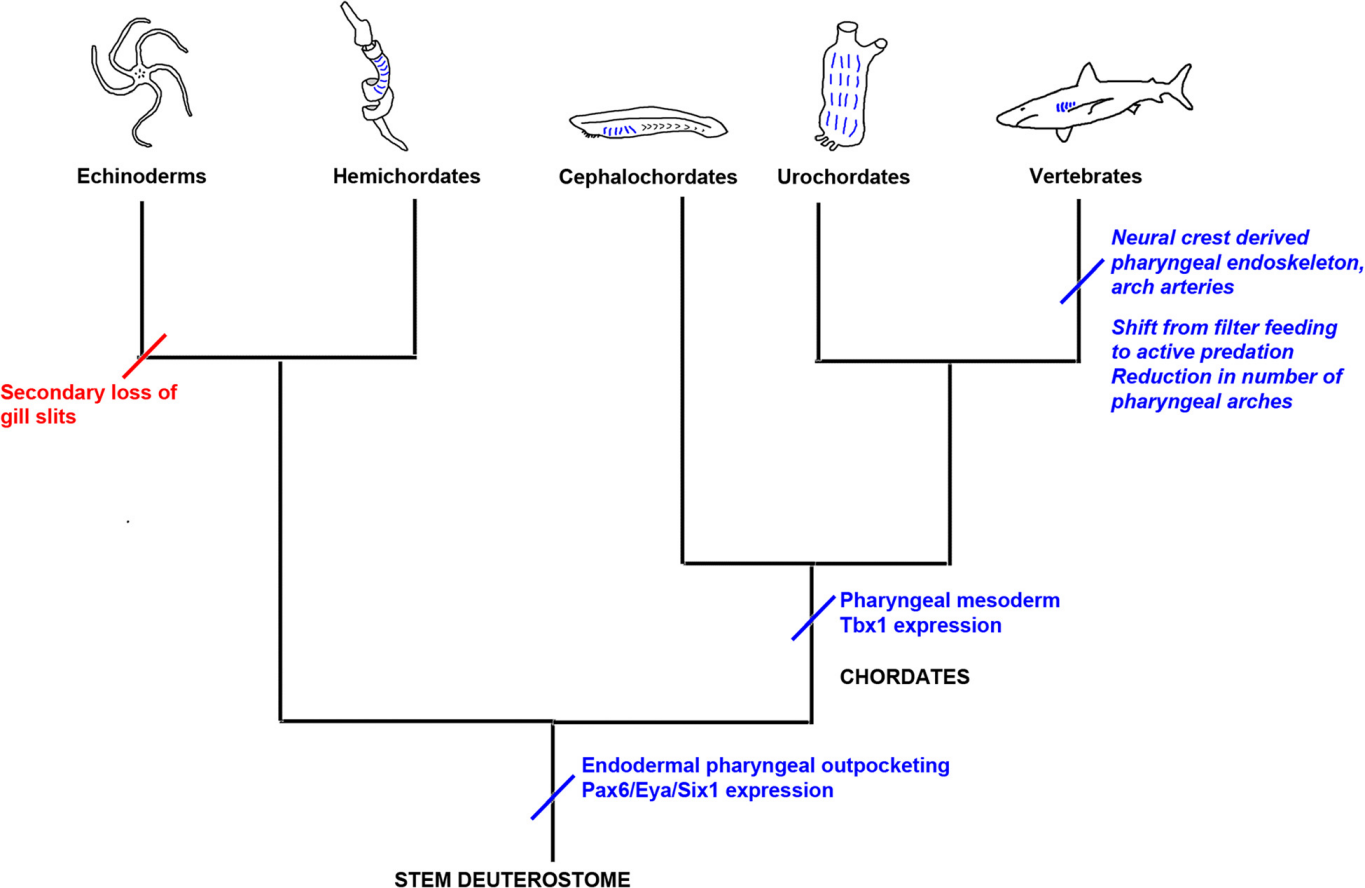
**Deuterostome phylogeny and the origin of pharyngeal segmentation.** Acquisition of characteristics is indicated in blue, loss in red. The proposed stem deuterostome is likely to have possessed pharyngeal slits. These have been secondarily lost in echinoderms but retained in the hemichordates. Within the chordate lineage, cephalochordates (for example, amphioxus) and urochordates retain pharyngeal slits in the adult form (indicated by blue lines). Within the vertebrate lineage, there was a shift away from filter feeding towards active predation. Modification to the pharyngeal segments included a reduction in number, a neural-crest-derived endoskeletal support, and arch arteries providing vasculature for the gills.

One can, therefore, conclude that pharyngeal development based around endodermal outpocketings is a primitive deuterostome feature and that the vertebrate pharyngeal arches are built around this ancient framework (Figure 3). The pharyngeal apparatus of vertebrates, however, differs significantly from that of other chordates. There are fewer pharyngeal segments in vertebrates and they are confined to a relatively small region behind the mouth. The vertebrate pharyngeal arches are also muscularized and have a neural-crest-derived cellular cartilaginous endoskeletal support. Developmentally, these alterations would lie in a reduction in the number of outpocketings generated by the endoderm and the infilling of the pharyngeal segments by neural crest and mesoderm, which, respectively, provide the skeleton and musculature of the arches. Evolutionarily, these changes would have been driven by the transition from filter feeding to a more predatory lifestyle with the origin of the vertebrates [13]. Notably, the primary function of the perforated pharynx would have shifted from filter feeding to respiration.

The neural-crest-derived cartilaginous endoskeleton of the pharynx clearly differentiates vertebrates from other chordates but it also important to appreciate that the endoderm plays a significant role in directing the development of the neural crest cells. These cells form a multipotent progenitor population that will generate a very broad range of derivatives; neurons, glia, melanocytes, cartilage, bone and connective tissue [23]. In the head, there is a correlation between the timing of migration and the subsequent fates of the neural crest cells [24]. The neural crest cells that populate the pharyngeal arches and generate skeletal derivatives are those that migrate early from the hindbrain, while those that migrate late, and do not enter the arches, stay close to the brain and form neurons and glia. However, it has been shown that there is no difference in potential between early- and late-migrating crest; late-migrating crest cells will form skeletogenic derivatives if they are directed to populate the pharyngeal arches [24]. Thus, the allocation of neural crest cells to form pharyngeal cartilage involves local cues within the arches and particularly those emanating from the epithelia. Correspondingly, in zebrafish mutants in which the endoderm fails to form, such as *bon* and *cas*, the neural-crest-derived pharyngeal cartilage fails to form [25]. It has also been shown that fibroblast growth factor (FGF) signalling plays key roles in directing neural crest cells to adopt an ectomesenchymal fate and the subsequent formation of cartilage [26,27]. Thus, the key to the development and evolution of the vertebrate pharyngeal arches is the establishment of endodermal outpocketing and subsequent epithelial influence on the fate of the neural crest cells that fill these segments to direct them to generate ectomesenchymal derivatives.

### From jawless to jawed vertebrates

Within the vertebrates, the first pharyngeal arch became modified to form the jaw; central to this was dorsoventral regionalization within the arches. In contrast to the pharyngeal skeleton of gnathostomes, which consists of separate dorsal and ventral elements connected by a joint, the lamprey pharyngeal skeleton consists of rods of cartilage that fuse to form an unjointed branchial basket [28,29]. Studies in gnathostomes have also shown that nested *Dlx* expression plays a central role in dorsoventral regionalization and that this is regulated by endothelin signalling [30]. Intriguingly, recent studies in lampreys have shown that aspects of dorsoventral regionalization are also present in lampreys. They display dorsoventrally restricted expression of *Dlx* genes, and other key transcription factors, and, as in gnathostomes, endothelin signalling is important for the ventral pharyngeal skeleton in lampreys, as it is in gnathostomes [28,29]. Thus, the evolution of the gnathostome jaw was built on a pre-existing DV patterning programme present in agnathans.

### Pharyngeal metamerism - beyond the phylotypic stage

**Figure 4:**
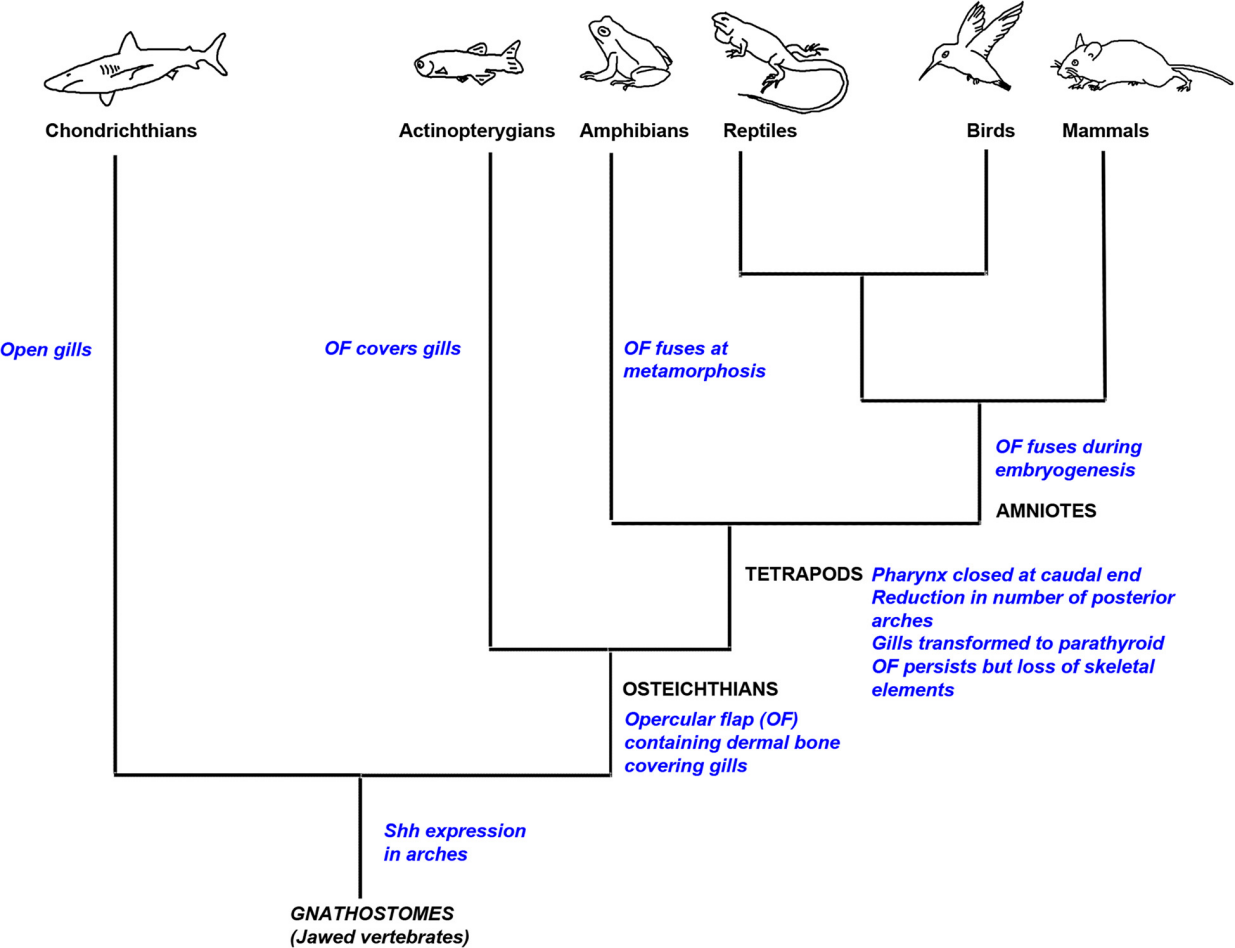
**Modification to the pharyngeal apparatus within the vertebrates.** Within the vertebrates, the pharyngeal region has undergone extensive modification. The chondrichthyans retain open gill slits, but in the actinopterygian fish, these are covered by a bony operculum, which is derived from the second arch. The tetrapods have undergone the most radical remodelling of the pharyngeal arches as part of their adaptation to terrestrial life. Within this grouping, amphibians possess an opercular flap that fuses at metamorphosis; in amniote embryos, the second arch still expands caudally to cover the posterior arches, but does not retain skeletal elements, and later fuses to the cardiac eminence. The internal gill buds have become modified to form the parathyroid gland.

A defining feature of the vertebrate phylotypic stages is the presence of the pharyngeal arches [31], and while the development of the pharyngeal apparatus is broadly similar within the vertebrates up to that point, divergent paths subsequently emerge (Figure 4). Within most chondrichthyans, the underlying embryonic pharyngeal segmentation is preserved in the external adult anatomy, as evidenced in the array of gills slits. In actinopterygians, however, the gills are not readily externally apparent but are covered by the operculum, a large flap that provides protection to the gills. Its movement helps to draw water into the pharynx and thus it plays critical roles in feeding and respiration. During development, the opercular bones form within the second arch and expand posteriorly to overlie the gill-bearing arches [32]. An operculum and gills are also present in some extant extant sarcopterigians, such as coelacanths and lungfish, but not in tetrapods. Fossil evidence, however, demonstrates that within the tetrapod stem group there was a stepwise loss of the operculum and gills. Thus while an operculum was found in *Panderichthys* it is not found in *Tiktaalik*, although this animal did possess a gill chamber [33-35].

### Tetrapod evolution - remodelling of the posterior pharyngeal segments

It was with the evolution of the tetrapods that the posterior pharyngeal arches as a whole underwent substantial remodelling. As the posterior arches no longer generated gill buds, and primary respiratory function shifted to the lungs, there was a reduction in arch number from seven to five, and this is the situation seen in amniotes, including human beings. A consequence of the loss of the operculum was that the posterior end of the pharynx no longer had an external opening. This also resulted in the internalization of the posterior pharyngeal arches and thus the overshadowing of the metameric origin of the pharynx in adult anatomy. The tetrapod transition, however, also required the emergence of new structures to facilitate life out of the water; prominent amongst these was the evolution of the parathyroid glands and parathyroid hormone [35]. Fish can take up calcium from the aquatic environment but this is not an option for tetrapods. Therefore, new controls for regulating calcium homeostasis had to be put in place. The parathyroids detect changes in the levels of calcium in the blood via the calcium-sensing receptor (CASR); if levels are low, they release parathyroid hormone (PTH) which acts to mobilize calcium release from internal stores such as bone and modulates renal ion transport. Recent developmental studies, however, have indicated that the evolution of the tetrapods did not involve as dramatic an alteration to the pharynx as the paleontological or anatomical evidence suggests.

Embryologically, the parathyroid gland is derived from the pharyngeal pouch endoderm, and it has been shown that its development is under the control of a key regulatory gene, *Gcm2*[36-38]. *Gcm2* is exclusively expressed in the parathyroid, and its embryonic anlagen, in mammals and avians; when this gene is mutated in mice, the parathyroid glands do not form. Although only tetrapods possess a parathyroid gland, it has been shown that the *Gcm2* gene is found throughout the gnathostomes and that in zebrafish and dogfish, this gene is also expressed in the pharyngeal pouches, and their derivatives, the internal gill buds [39]. Studies in zebrafish have further demonstrated that *Gcm2* is required for the elaboration of the gill buds from the pharyngeal pouches. Furthermore, while it was generally believed that fish not only lack the parathyroid glands but also PTH, more recent work has shown that PTH-encoding genes are present in teleosts [39-41]. *PTH* has been found to be expressed in the gills, as has the *CASR* gene. These facts clearly suggest that the internal gills of fish and the parathyroid glands are related structures that share a common evolutionary origin. Both rely on *Gcm2* for their development, and both express key components of the regulatory apparatus for controlling extracellular calcium levels. Thus with the evolution of the tetrapods, the gills were not lost but rather were transformed into the parathyroid glands [39].

There are also a number of reasons to believe that the opercular flap was not completely lost during tetrapod evolution but that it persists as an embryonic entity and is important in internalizing the posterior pharyngeal arches. Although, dermal ossifications - such as the opercle, found in actinopterygians, do not form in the second arch in amniotes, in both groups the development of the second arch is characterized by its disproportionate posterior expansion, whereby it comes to overlie the posterior arches, which form internal gills in fish or the parathyroids in amniotes (Figure 2) [3]. Furthermore, the second arches of both chick and zebrafish embryos express the same set of genes [3]. In particular, the caudal edge expresses *Shh*, which is a proliferative driver in many epithelia, and *Shh* signalling is required for posterior expansion of the second arch in both species [3].

Amniotes differ, however, from teleosts in that the posterior edge of the second arch does not remain open. Rather, it fuses with the cardiac eminence, which results in the posterior arches becoming enclosed in a cavity, the cervical sinus of His, which eventually becomes obliterated by the apposition and fusion of its walls, yielding the smooth contour to the external surface of the neck (Figure 2) [2]. These events mirror what is observed during amphibian metamorphosis and it has recently been shown that the fusion of the caudal edge of the second arch and the loss of the sinus also requires thyroid hormone signalling [3]. Chick embryos treated with antagonists of thyroid signalling display only partial fusions of the caudal edge of the second arch with the subjacent epithelia and the expansion and persistence of the sinus. Thus, in both amphibians and amniotes, the internalization of the gills and the eradication of the posterior external opening are homologous events driven by thyroid hormone.

## Conclusions

The development of the amniote pharyngeal apparatus is an intriguing process in that its phylogenetic history is readily discernible and insights into its stepwise assembly can be uncovered. We can detect the deuterostome origins of this programme, in the generation of endodermal outpocketing, around which the rest of its development is orchestrated. Another key feature of the development of this structure is the infilling of these segments by neural crest cells and their subsequent differentiation to form the endoskeletal support of the pharynx. This facet will have evolved with the vertebrates and is driven by interplay between the pre-existing epithelial segments with the multipotent neural crest cells. Beyond the phylotypic stage, the amniote pharynx becomes extensively remodelled. We can see here the replay of events associated with the evolution of the bony fish; the covering of the posterior arches by the opercular flap, and the evolution of the tetrapods; the internalization of the gills and the closure of the posterior opening of the gill chamber. However, it was found that the operculum or gills were not totally lost; rather that both persist. The gills have been transformed into the parathyroid glands of tetrapods and the operculum exists as an embryonic entity, never generating any bony elements. Thus, the development of the pharynx has been profoundly shaped by its evolutionary history.

## Abbreviations

CASR: calcium in the blood via the calcium-sensing receptor; FGF: fibroblast growth factor; PTH: parathyroid hormone.

## Competing interests

The authors declare that they have no competing interests.

## Authors’ contributions

AG and JR wrote the article. Both authors read and approved the final manuscript.
